# Differences between perceived and recommended nutritional needs among three division I female sports teams

**DOI:** 10.1080/15502783.2025.2550210

**Published:** 2025-08-28

**Authors:** Shelley L. Holden, Matthew T. Stratton, Austin T. Massengale, Ray Davis, Jarrett Strate-Lutzow, Kaitlyn Evenson-McMurtry

**Affiliations:** University of South Alabama, Basic and Applied Laboratory for Dietary Interventions in Exercise and Sport, Department of Health, Kinesiology & Sport, Mobile, AL, USA

**Keywords:** Sport nutrition, energy expenditure, dietary needs, athletes

## Abstract

**Background:**

Nutritional intake can greatly impact an athlete’s ability to adequately perform the necessary demands of their sport. However, previous literature has demonstrated that many athletes lack the nutritional knowledge necessary to follow the recommended nutritional guidelines for their activity level. Whether this differs by sport has yet to be elucidated. Therefore, the purpose of this investigation was to assess the differences between perceived and recommended nutritional needs in NCAA Division I female athletes across three sports: soccer, softball, and volleyball

**Methods:**

Fifty-six female NCAA Division I athletes (soccer: *n* = 23; softball: *n* = 14, and volleyball: *n* = 19) completed a survey assessing their perception regarding recommended daily intakes for total kcals, proteins (PRO), carbohydrates (CHO), and fats (F). These perceived needs were then compared to the recommended guidelines set forth by the American College of Sports Medicine (ACSM) and the International Society of Sports Nutrition (ISSN) for low, medium, and high levels of daily activity. Difference scores between perceived and recommended intakes were calculated and a repeated measures ANOVA was conducted to examine discrepancies between athletes’ perceived daily kcal needs and individualized recommendations based on low, moderate, and high activity levels.

**Results:**

A significant main effect of activity level on kcal discrepancy was seen (*p* < .001, η^2^ *p* = 0.983) indicating that differences between perceived and recommended intake varied strongly by activity level. A significant main effect of sport was also found (*p* = .007, η^2^ *p* = 0.173) showing that athletes’ estimates differed by sport. A significant interaction between activity level and sport was found (*p* = .007, η^2^ *p* = 0.173) indicating that the degree of over- or underestimation varied by sport across the activity levels. Post hoc comparisons revealed that soccer athletes’ perceived kcal needs were more closely aligned with moderate and high activity level recommendations, whereas volleyball and softball athletes significantly underestimated their needs, particularly at higher activity levels. No significant differences were observed between sports at the low activity level. Similar patterns were observed for PRO, CHO, and F intake, with strong main effects for activity level (all *p* < .001, η^2^ *p* ≈ 0.98), significant sport effects (*p* = .007), and activity level × sport interactions (*p* = .006–.007).

**Conclusions:**

Overall, the findings suggest a need for targeted nutrition education in sports where athletes tend to underestimate their dietary requirements.

